# Group B Streptococcus (GBS) Colonization and Disease among Pregnant Women: A Historical Cohort Study

**DOI:** 10.1155/2019/5430493

**Published:** 2019-02-03

**Authors:** James M. Edwards, Nora Watson, Chris Focht, Clara Wynn, Christopher A. Todd, Emmanuel B. Walter, R. Phillips Heine, Geeta K. Swamy

**Affiliations:** ^1^Maternal Fetal Medicine, WakeMed Health and Hospitals, Raleigh, NC, USA; ^2^Division of Maternal-Fetal Medicine, Department of Obstetrics and Gynecology, Duke University Medical Center, Durham, NC, USA; ^3^The Emmes Corporation, Rockville, MD, USA; ^4^Duke Human Vaccine Institute, Duke University Medical Center, Durham, NC, USA; ^5^Department of Pediatrics, Duke University Medical Center, Durham, NC, USA

## Abstract

**Background:**

Maternal GBS colonization is associated with early-onset neonatal sepsis and extensive efforts are directed to preventing this complication. Less is known about maternal risks of GBS colonization. We seek to provide a modern estimate of the incidence and impact of maternal GBS colonization and invasive GBS disease.

**Methods:**

A single center historical cohort study of all births between 2003 and 2015 was performed. Data was collected via electronic health record abstraction using an institutional specific tool. Descriptive statistics were performed regarding GBS status. Inferential statistics were performed comparing risk of adverse pregnancy outcomes in cohorts with and without GBS colonization as well as cohorts with GBS colonization and invasive GBS disease.

**Results:**

A total of 60,029 deliveries were included for analysis. Overall, 21.6% of the population was GBS colonized and 0.1% had invasive GBS disease. GBS colonization was associated with younger maternal age, Black race, non-Hispanic ethnicity, chronic hypertension, preexisting diabetes, and tobacco use (p<0.01). In the adjusted analyses, there was an increased risk of gestational diabetes (aRR 1.21, 95% CI 1.11-1.32) in colonized pregnancies and a decreased incidence of short cervix (aRR 0.64, 95% CI 0.52-0.79), chorioamnionitis (aRR 0.76, 95% CI 0.66-0.87), wound infection (aRR 0.75, 95% CI 0.64-0.88), and operative delivery (aRR 0.85, 95% CI 0.83-0.88).

**Conclusions:**

This modern-day large cohort of all births over a 12-year period demonstrates a GBS colonization rate of 21.6%. This data reflects a need to assess maternal and perinatal outcomes in addition to neonatal GBS sepsis rates to inform decisions regarding the utility of maternal vaccination.

## 1. Introduction

Pregnancy associated group B streptococcus (GBS) is a well-established cause of significant neonatal morbidity and mortality [1]. Consequently, extensive efforts are directed towards the prevention of this devastating infection [2]. Current recommendations focus on maternal GBS screening and intrapartum prophylactic antibiotics to prevent early-onset neonatal infection [3]. Recent renewed efforts to prevent both early- and late-onset neonatal infection have focused on maternal vaccination against GBS in order to provide neonatal benefit [4, 5]. Conversely, few systematic studies exist regarding the potential maternal or pregnancy-related benefits of vaccination [6].

Maternal GBS colonization and infection have been associated with numerous adverse outcomes such as higher rates of febrile morbidity [7] and chorioamnionitis [8, 9], which are associated with maternal sepsis [10], endometritis [11], cesarean delivery [11], and postoperative wound infections [12]. Maternal GBS bacteriuria is associated with pyelonephritis and other ascending infections which can lead to maternal sepsis as well as preterm birth [13, 14]. Additionally, GBS can cause maternal mastitis and breast abscess which have been associated with significant maternal morbidity [15, 16]. However, much of the data on maternal GBS infection is limited to sporadic case reports and case series, which limits the ability to reliably estimate the impact of GBS infection on maternal morbidity. Therefore, a systematic investigation of maternal GBS colonization and GBS invasive disease can provide evidence to support the potential maternal benefits and improved obstetric outcomes from a GBS vaccine administered during pregnancy.

We hypothesized that GBS colonization is associated with an increase in adverse maternal and pregnancy outcomes. The primary objectives of this study were to estimate the prevalence of GBS colonization, compare the risk of adverse pregnancy outcomes by GBS colonization status, and estimate the incidence of invasive GBS disease. Secondary objectives included exploring the sites of GBS invasive disease and identifying pregnancy and maternal factors associated with colonization and invasive disease, thereby improving our understanding of the infection-related maternal morbidity associated with GBS colonization.

## 2. Materials and Methods

This historical cohort study was planned and prepared using the STROBE guidelines [17]. Ethical and protocol review and approval were performed by the Duke Health Institutional Review Board prior to study initiation (Pro00068085). Electronic data abstraction was performed using the Duke Enterprise Data Unified Content Explorer (DEDUCE) system [18]. This online tool empowers investigators with real-time access to deidentified information collected as a byproduct of patient care. DEDUCE compiles its data from multiple source systems and allows the researcher to filter through millions of rows of data to define a clinical cohort for study.

All pregnant women who delivered at a Duke Health affiliated hospital between January 1, 2003 and December 31, 2015 were eligible for inclusion. GBS status was defined by International Classification of Diseases, 9th and 10th revisions (ICD-9 and ICD-10) diagnostic codes obtained from inpatient and outpatient visits during pregnancy through the delivery visit (Supplemental Table 1). To estimate the prevalence of GBS, subjects were classified as GBS colonized or not colonized based on results from ICD-9/ICD-10 coding for GBS carrier status at any gestational age. Rectovaginal culture or urine culture results in a proportion of patients were compared to ICD coding in order to assess accuracy. Subjects without positive culture results or ICD-9/ICD-10 coding for GBS carrier status were presumed not colonized. Invasive GBS disease was classified per the Centers for Disease Control and Prevention (CDC) guidelines as isolation of GBS from a normally sterile site, such as blood, cerebrospinal fluid (CSF), pleural fluid, peritoneal fluid, pericardial fluid, bone, joint/synovial fluid, placenta, amniotic fluid, or other internal body site [19]. Demographic characteristics and history of tobacco use were obtained from antenatal outpatient visit records. ICD-9/ICD-10 diagnostic codes were used to identify chronic health conditions (hypertension, preexisting diabetes, autoimmune disorder, and chronic infectious disease), adverse pregnancy outcomes (gestational diabetes, preeclampsia, and preterm labor), and infectious complications (chorioamnionitis, endometritis, pyelonephritis, mastitis and sepsis), reported during pregnancy through delivery.

Demographic characteristics and putative risk factors were compared by GBS colonization or GBS disease status using t-tests for continuous measures and Chi-squared tests or Fisher's exact tests for categorical measures. Significance levels were reported for these unadjusted comparisons of characteristics in GBS colonized versus GBS negative women and in invasive GBS cases versus GBS colonized women. To evaluate for independent predictors of GBS colonization, all demographic and risk factors were included as independent variables in a multivariable logistic regression model of the GBS colonized versus GBS negative women. Characteristics that were independently associated with GBS colonization at a significance level of <0.05 or identified as known confounders were included as covariates in multivariate models of GBS colonization status. Relative risks of adverse outcomes were estimated using separate Poisson regression models of GBS colonized versus GBS negative women, unadjusted and adjusted for demographic characteristics and potential confounders. Robust standard errors were specified as appropriate for the estimation of relative risk for binary data [20]. Incidence of adverse pregnancy outcomes was compared in invasive GBS versus GBS colonized women using Fisher's exact test as appropriate to the small number of events in the invasive GBS group. Among women with invasive GBS, frequencies of sites of GBS infection were reported and clinical characteristics or outcomes described. All analyses were conducted using SAS 9.4.

## 3. Results

### 3.1. GBS Colonization and Invasive Disease Prevalence

Using the DEDUCE system we identified 60,029 pregnant pregnancies which delivered at a Duke Health affiliated hospitals between January 1, 2003, and December 31, 2015. A total of 47,013 (78.3%) pregnancies were determined to be GBS negative, 12,952 (21.6%) pregnancies were determined to be GBS colonized, and 64 (0.1%) pregnancies were found to be complicated by invasive GBS disease.

### 3.2. Risk Factors for GBS Colonization and Invasive Disease

Demographic factors and clinical characteristics were explored in relation to GBS colonization and invasive disease status as described in Table 1. First, the GBS colonized and GBS negative pregnancies were compared with several significant differences (Table 1). Mean observed maternal age at delivery was lower among GBS colonized than GBS negative women (28.70 ± 6.2 versus 28.07 ± 6.2, p<0.001). GBS colonized women were more likely than GBS negative women to be Black or African American (45.7% versus 31.6%, p<0.001) and less likely to be Hispanic (11.8% versus 16.8%, p<0.001). They were also more likely to be exposed to antibiotics prior to delivery (9.8% versus 3.4%, p<0.001). Finally, GBS colonized women were more likely to have chronic hypertension (8.6% versus 7.2%, p<0.001), preexisting diabetes (3.5% versus 2.8%, p<0.001), and history of tobacco use (20.8% versus 17.8%, p<0.001). In a multivariable model to evaluate predictors of GBS colonization, maternal age at delivery (aRR 0.99, 95% CI 0.99-0.99), Black or African American race (aRR 1.48, 95% CI 1.41-1.54), and preexisting diabetes (aRR 1.12, 95% CI 1.01-1.23) were independently associated with risk of GBS colonization (Table 2).

When the population of women with invasive GBS disease was compared to the GBS colonized women (Table 1), there were no significant differences in race, ethnicity, or antibiotic exposure between groups. Women with invasive GBS disease were more likely than GBS colonized women to have chronic hypertension (25.0% versus 8.6%, p<0.001) and preexisting diabetes (15.6% versus 3.5%, p<0.001). Multivariate analysis for the comparison of invasive disease and GBS colonized cohorts was not possible due to the low number of invasive GBS disease cases.

### 3.3. Adverse Pregnancy Outcomes in GBS Colonization and Invasive Disease

In adjusted comparisons of GBS colonized and GBS negative women, there was an increased risk of gestational diabetes (aRR 1.21, 95% CI 1.11-1.32) in colonized pregnancies (Table 3). However, there was a decreased risk of short cervix (aRR 0.64, 95% CI 0.52-0.79), chorioamnionitis (aRR 0.76, 95% CI 0.66-0.87), wound infection (aRR 0.75, 95% CI 0.64-0.88), and operative delivery (aRR 0.85, 95% CI 0.83-0.88). There was also a decreased risk of preterm birth at <34 (aRR 0.30, 95% CI 0.26-0.35) and <37 weeks gestation (aRR 0.49, 95% CI 0.45-0.53). There were no significant differences in gestational hypertension, preterm labor, endometritis, pyelonephritis, mastitis or sepsis in the multivariate analysis.

Adverse pregnancy outcomes were also explored in comparing the invasive GBS cohort and the GBS colonized cohort (Table 3). In unadjusted analyses, women with invasive GBS disease were more likely to have gestational diabetes, preterm labor, preterm birth, short cervix, chorioamnionitis, endometritis, sepsis, and wound infections (all p<0.05) versus GBS colonized women. Adjusted analyses were not performed due to the low number of GBS invasive cases. Finally, the invasive GBS cohort was evaluated to determine the site of GBS infection (Table 4). The predominant sites from which invasive GBS was cultured were the cervix and genital area. These sites were included in analysis of invasive disease as cultures were all collected from women with symptomatic infection in these areas. The remainder represented sites from across the body.

## 4. Discussion

This large modern-day cohort indicates that approximately 1 in 5 pregnancies are complicated by GBS colonization. Importantly, we identified adverse pregnancy outcomes associated with GBS colonization such as gestational diabetes. These findings point towards potential maternal and pregnancy benefits of GBS vaccination.

The finding of increased gestational diabetes in the GBS colonized cohort compared to the GBS negative cohort is new. While this finding may be due to chance or represent some unmeasured confounder associated with this specific population, this finding may represent that the immunologic impairment associated with GDM increases the risk of GBS colonization. However, previous evidence demonstrates that there is not an increase in GBS colonization in the GDM population [21]. Conversely, it is plausible that GBS colonization is associated with an otherwise subclinical inflammatory response. Development of gestational diabetes is associated with increased systemic inflammation [22]. Additionally, infection induced inflammation induced by periodontitis is associated with an increased risk for gestational diabetes [23]. This indicates that GBS vaccination may need to be administered early in pregnancy or even preconception in order to receive maximum benefit for systemic inflammation driven processes.

In this cohort, GBS colonized women were generally less healthy than GBS negative women, as indicated by increased proportion having chronic hypertension, preexisting diabetes, and history of tobacco use. Furthermore, Black or African American women were overrepresented in the GBS colonized group while conversely, Hispanic ethnicity was underrepresented. This may promote existing racial and ethnic disparities in adverse pregnancy outcomes [24].

Interestingly, some adverse outcomes were less common among GBS colonized women in comparison to GBS negative women. Short cervix, chorioamnionitis, wound infection, preterm birth, and operative delivery rates were decreased in the GBS colonized population. This is different from previously published studies [8, 9, 12]. These earlier studies predate the modern GBS screening and treatment algorithms. Following updated guidelines for the prevention of early-onset neonatal infection in 2002, GBS colonized women routinely receive prophylactic intravenous antibiotics during labor [2]. If colonization is identified by positive urine culture, a treatment course of antibiotics is routinely prescribed. The resulting reduction in bacterial burden likely decreases the incidence of chorioamnionitis and wound infection rates and may decrease the risk of short cervix associated with subclinical infection. Preterm birth rates among GBS positive women are likely artificially reduced as many women who deliver preterm will not undergo standard GBS screening at 36 weeks and therefore may be classified as GBS negative. Finally, operative delivery rates may be decreased secondary to decreased chorioamnionitis rates which serves as a common indication to expedite delivery. In combination, these findings point to potential benefit of antibiotic treatment for GBS prophylaxis beyond that associated with the neonate. This will need to be accounted for when considering the benefits of GBS vaccination and whether guidelines include both vaccination and continued antibiotic prophylaxis.

Additionally, pregnancies with invasive GBS disease were investigated. In comparison to women with GBS colonization, these women had more preexisting conditions and increased rates of adverse pregnancy outcomes. Here infectious complications were more common as opposed to the comparison between GBS colonized and GBS negative groups. This likely reflects that invasive GBS disease is commonly defined based on GBS culture from obstetric related complications such as cervical culture, wound culture, amniotic fluid culture, and blood cultures.

The primary strength of this study is the large, comprehensive cohort. All pregnancies in our health system during a 13-year contemporary period were included. The electronic abstraction system allowed complete chart abstraction for all pregnancies. Additionally, many of the variables were abstracted directly from laboratory and culture records reducing the rate of coding errors. The primary limitation to this study is the historical nature of the data. Data collection is dependent on the accuracy of the medical record at the time of patient care. Furthermore, several data points were abstracted from hospital charges which may not always represent actual clinical care. Finally, the low number of patients receiving inpatient antibiotics prior to delivery, particularly in the GBS colonized group raises some concern about the abstraction of pharmacy records. This limits the ability to use antibiotic exposure as a subanalysis factor as planned in the initial study protocol.

## 5. Conclusions

When an efficacious GBS vaccine is developed and approved, these findings indicate a need to assess the maternal effects of transitioning to a GBS vaccination strategy for neonatal GBS disease prevention as there may be additional benefits to intrapartum antibiotic prophylaxis beyond neonatal disease prevention.

## Figures and Tables

**Table 1:** Demographic and clinical characteristics by GBS status.

	**GBS Negative**	**GBS Colonized**	**Invasive GBS**	**P Value** ^**1**^	**P Value** ^**2**^
	**(N=47,013)**	**(N=12,952)**	**(N=64)**		
**Age at delivery (years)**					
Mean (SD)	28.7 (6.2)	28.0 (6.2)	28.1 (6.4)	<.001	0.95
**Race, n (**%**)**					
Black or African American	14012/44293 (31.6%)	5660/12379 (45.7%)	39/64 (60.9%)	<.001	0.05
White or Caucasian	20325/44293 (45.9%)	4740/12379 (38.3%)	19/64 (29.7%)	-	-
Other	9956/44293 (22.5%)	1979/12379 (16.0%)	6/64 (9.4%)	-	-
**Ethnicity, n (**%**)**					
Hispanic	7506/44657 (16.8%)	1467/12443 (11.8%)	2/63 (3.2%)	<.001	0.08
Not Hispanic	35580/44657 (79.7%)	10611/12443 (85.3%)	58/63 (92.1%)	-	-
Other	1571/44657 (3.5%)	365/1244 (2.9%)	3/63 (4.8%)	-	-
**Antibiotic use**					
Inpatient antibiotic administered	1590/47013, 3.4%	1271/12952, 9.8%	3/64, 4.7%	<0.001	0.17
during pregnancy prior to delivery					
**Risk factors, n (**%**)**					
Chronic hypertension	3366/47013 (7.2%)	1112/12952 (8.6%)	16/64 (25.0%)	<0.001	<.001
Pre-existing diabetes	1294/47013 (2.8%)	456/12952 (3.5%)	10/64 (15.6%)	<0.001	<.001
Autoimmune disorder	310/47013 (0.7%)	72/12952 (0.6%)	0/64 (0%)	0.19	1.00
Chronic infectious disease^3^	368/47013 (0.8%)	106/12952 (0.8%)	1/64 (1.6%)	0.69	0.41
Tobacco use	5887/33129 (17.8%)	2010/9674 (20.8%)	14/51 (27.5%)	<0.001	0.23

^1^P value for comparison of GBS negative versus GBS colonized.

^2^P value for comparison of GBS colonized versus invasive GBS.

^3^Human immunodeficiency virus, hepatitis B, and hepatitis C.

P value from t-test or Chi-square or Fisher's exact test.

**Table 2:** Multivariate predictors of GBS colonization.

**Characteristic**	**aRR**	95%** CI**	**P value**
Maternal age at delivery (years)	0.99	(0.99, 0.99)	<0.001
Race^1^			
Black or African American	1.48	(1.41, 1.54)	<0.001
Other	0.92	(0.85, 1.00)	0.06
Ethnicity^2^			
Hispanic	0.94	(0.85, 1.03)	0.20
Other	0.79	(0.59, 1.06)	0.12
Chronic hypertension	1.03	(0.96, 1.09)	0.44
Pre-existing diabetes	1.12	(1.01, 1.23)	0.03
Autoimmune disorder	0.78	(0.61, 1.01)	0.06
Tobacco use	1.01	(0.96, 1.05)	0.83

^1^Reference = White or Caucasian.

^2^Reference = not Hispanic.

aRR = adjusted relative risk.

CI = confidence interval.

P value from a multivariable generalized linear model of all characteristics as independent variables and GBS colonization versus GBS negative as the dependent variable.

**Table 3:** Incidence and relative risk of adverse pregnancy outcomes by GBS colonization status.

	**GBS Negative**	**GBS Colonized**	**Invasive GBS**		
	**(N=47,013),**	**(N=12,952),**	**(N=64),**		
**Outcome**	**n (**%**)**	**n (**%**)**	**n (**%**)**	**RR (95% CI)**	**aRR** ^**1**^ ** (95% CI)**
Gestational diabetes	1515 (3.2%)	559 (4.3%)	12 (18.8%)^+^	1.34 (1.22, 1.47)	1.21 (1.11, 1.32)
Gestational hypertension/preeclampsia	5397 (11.5)	1585 (12.2%)	9 (14.1%)	1.07 (1.01, 1.12)	0.98 (0.93, 1.04)
Pyelonephritis	412 (0.9%)	140 (1.1%)	1 (1.6%)	1.23 (1.02, 1.49)	1.12 (0.90, 1.38)
Short cervix	760 (1.6%)	172 (1.3%)	7 (10.9%)^+^	0.82 (0.70, 0.97)	0.64 (0.52, 0.79)
Preterm labor	5528 (11.8%)	1532 (11.8%)	15 (23.4%)^+^	1.01 (0.95, 1.06)	0.96 (0.90, 1.02)
Preterm Birth, < 34 weeks	2812 (6.2%)	273 (2.2%)	11 (17.7%)^+^	0.35 (0.31, 0.40)	0.30 (0.26, 0.35)
Preterm Birth, < 37 weeks	6550 (14.4%)	1003 (8.0%)	20 (32.3%)	0.55 (0.52, 0.59)	0.49 (0.45, 0.53)
Chorioamnionitis	1654 (3.5%)	386 (3.0%)	10 (15.6%)^+^	0.85 (0.76, 0.94)	0.76 (0.66, 0.87)
Operative delivery	17354 (36.9%)	4088 (31.6%)	27 (42.2%)	0.85 (0.83, 0.88)	0.85 (0.83, 0.88)
Wound infection	1161 (2.5%)	257 (2.0%)	9 (14.1%)^+^	0.80 (0.70, 0.92)	0.75 (0.64, 0.88)
Endometritis	142 (0.3%)	50 (0.4%)	3 (4.7%)^+^	1.28 (0.93, 1.76)	1.03 (0.72, 1.49)
Sepsis	41 (0.1%)	15 (0.1%)	2 (3.1%)^+^	1.33 (0.74, 2.40)	1.45 (0.74, 2.85)
Mastitis	82 (0.2%)	31 (0.2%)	0	1.37 (0.91, 2.07)	1.12 (0.69, 1.82)

% = n/column N.

RR = relative risk, aRR = adjusted relative risk, and CI = confidence interval.

RR and aRR reported for GBS colonized versus GBS negative.

^1^Adjusted for age, race, chronic hypertension, preexisting diabetes, and tobacco use.

^+^Fisher's exact p <0.05 for comparison of invasive GBS versus GBS colonized.

**Table 4:** Frequency of invasive GBS by specimen source.

	**Invasive GBS**	
	**(N=64) (0.11**%**)**	
**Specimen Source**	**n**	%
Cervix/Genital	33	51.6
Placenta	5	7.8
Amniotic fluid	4	6.3
C-section site	4	6.3
Abdominal	3	4.7
Blood	3	4.7
Thigh	3	4.7
Buttocks	2	3.1
Leg	2	3.1
Abscess - Site Unknown	1	1.6
Foot	1	1.6
Pelvic	1	1.6
Stomach	1	1.6
Throat	1	1.6

## Data Availability

The deidentified data used to support the findings of this study are available from the corresponding author upon request.

## Supplementary Materials

Supplementary Materials Supplemental Table 1: ICD-9 and -10 codes for data abstraction.
