# Supplementary material for: Group B Streptococcus (GBS) Colonization and Disease among Pregnant Women: A Historical Cohort Study
Source: Infect Dis Obstet Gynecol. 2019 Feb 3;2019:5430493. doi: 10.1155/2019/5430493 (PMC6378061; doi:10.1155/2019/5430493)
Supplement: Supplementary Materials — Supplemental Table 1: ICD-9 and -10 codes for data abstraction. [file 5430493.f1.pdf]

Supplemental Table 1. ICD-9 and -10 Codes for Data Abstraction.

	ICD Codes
GBS Colonization	Z22.330, V02.51
Pre-existing Diabetes	250,E08,E08.43,E08.36,E08.22,E08.620,"E08.41, E08.21, E08.610, E08.40, E08.52, E08.42, E08.621, E08.65, E08.01, E08.00, E08.649, E08.11, E08.10, E08.321, E08.329, E08.331, E08.339, E08.59, E08.618, E08.29, E08.49, E08.39, E08.628, E08.622, E08.69, E08.9, E08.630, E08.351, E08.359, E08.341, E08.349, E08.8, E08.311 ,E08.319, 250.0, 250.2, 250.1, 250.6, 250.5, 250.8, 250.7, 250.4, 250.9, E09, E09.22, E09.21, E09.610, E09.621, E09.65 , E09.00, E09.64, E09.649, E09.11, E09.10, E09.321, E09.331, E09.43, E09.40, E09.42, E09.49, E09.59, E09.29, E09.39, E09.628, E09.9, E09.351, E09.359, E09.8, E09.319, O24.811, O24.812, O24.813, O24.819, E13.44, E13.43, E13.36, E13.22, E13.21, E13.610, E13.40, E13.52, E13.51, E13.42, E13.621, E13.65, E13.01, E13.00, E13.649, E13.11, E13.10, E13.321, E13.329, E13.331, E13.339, E13.59, E13.618, E13.29, E13.49, E13.39, E13.628, E13.622, E13.69, E13.9, E13.351, E13.359, E13.341, E13.8, E13.311, E13.319, O24.02, O24.011, O24.012, O24.013, O24.019, O24.03, O24.12, O24.111, O24.112, O24.113, O24.119, O24.13, 249.20, 249.21, 249.10, 249.11, 249.6, 249.60, 249.61, 249.5, 249.50, 249.51, 249.30, 249.80, 249.81, 249.00, 249.01, 249.70, 249.71, 249.4, 249.40, 249.41, 249.90, 249.91, E10, E10.44, E10.43, E10.36, E10.22, E10.41, E10.21, E10.610, E10.40, E10.52, E10.51, E10.42, E10.621, E10.65, E10.64, E10.641, E10.649, E10.1, E10.11, E10.10, E10.321, E10.329, E10.331, E10.339, E10.59, E10.618, E10.29, E10.49, E10.39, E10.628, E10.622, E10.69, E10.9, E10.351, E10.359, E10.341, E10.349, E10.8, E10.311, E10.319, E11, E11.44, E11.43, E11.36, E11.22, E11.620, E11.41, E11.21, E11.610, E11.40, E11.52, E11.51, E11.42, E11.621, E11.65, E11.01, E11.00, E11.64, E11.641, E11.649, E11.321, E11.329, E11.331, E11.339, E11.4, E11.59, E11.618, E11.29, E11.49, E11.39, E11.638, E11.628, E11.622, E11.69, E11.9, E11.630, E11.351, E11.359, E11.341, E11.349, E11.8, E11.311, E11.319, 250.20, 250.22, 250.10, 250.12, 250.60, 250.62, 250.50, 250.52, 250.30, 250.32, 250.80, 250.82, 250.00, 250.02, 250.70, 250.72, 250.40, 250.42, 250.90, 250.92, 250.21, 250.23, 250.11, 250.13, 250.61, 250.63, 250.51, 250.53, 250.31, 250.33, 250.81, 250.83, 250.01, 250.03, 250.71, 250.73, 250.41, 250.43, 250.91, 250.93, O24.32, O24.311, O24.312, O24.313, O24.319, O24.33
Chronic Hypertension	642.03, 642.00, 642.04, 642.01, 642.02, 401, 401.1, 401.0, I10, I15.2, I15.1, 642.13, 642.10, 642.14, 642.11, 642.12, 642.23, 642.20, 642.24, 642.21, 642.22, 405.19, I15.8, 405.09, 405.99, I27.2, O10.02, O10.011, O10.012, O10.013, O10.019, O10.03, O11.2, O11.3, O11.9, O10.42, O10.413, 405, 405.1, I15, I15.9, 401.9, O10.92, O10.911, O10.912, O10.913, O10.919, O10.93"
Autoimmune Disorder	M08.272, M08.222, M08.262, M08.232, M08.2, M08.29, M08.261, M08.231, M08.20, M08.3, 714.33, M05.872, M05.822, M05.842, M05.852, M05.862, M05.812, M05.832, M05.89, M05.871, M05.821, M05.841, M05.851, M05.861, M05.811, M05.831, M05.879, M05.829, M05.849, M05.869, M05.80, M05.839, 714.2, M06.872, M06.822, M06.842, M06.852, M06.862, M06.832, M06.89, M06.871, M06.821, M06.841, M06.861, M06.811, M06.831, M06.859, M06.869, M06.80, 714.32, M08.472, M08.422, M08.442, M08.462, M08.432, M08.4, M08.471, M08.421, M08.441, M08.461, M08.40, 714.31, 714.30, 714.0, 714, M05.642, M05.69, M06.9, M06.072, M06.022, M06.042, M06.052, M06.062, M06.012, M06.032, M06.0, M06.09, M06.071, M06.021, M06.041, M06.051, M06.061, M06.011, M06.031, M06.079, M06.049, M06.059, M06.069, M06.019, M06.00, M06.039, M06.08, M05, M05.772, M05.722, M05.742, M05.752, M05.762, M05.712, M05.732, M05.79, M05.771, M05.721, M05.741, M05.751, M05.761, M05.711, M05.731, M05.779, M05.729, M05.749, M05.769, M05.70, M05.739, M05.9, M06.212, M05.30, 714.81, M05.1, M05.19, M05.10, M05.471, M06.372, M06.322, M06.342, M06.362, M06.3, M06.371, M06.321, M06.341, M06.361, M06.329, M06.30, M05.59, M05.50, M05.2, M05.29, M05.211, M05.20, M08.022, M08.062, M08.032, M08.09, M08.00, M08.071, M08.021, M08.051, M08.031, L93.0, 373.34, H01.12, H01.124, H01.123, H01.129, M32.11, M32.14, M32.13, D68.62, 695.4, L93, M32.8, L93.2, M32.19, M32.12, L93.1, 710.0, M32.10, M32.9, M32.15, D68.61, 556.8, K51.8, K51.814, K51.813, K51.812, K51.818, K51.80, K51.811, K51.819, 556, K51, 556.9, K51.9, K51.913, K51.912, K51.918, K51.90, K51.911, K51.919, K50.8, K50.814, K50.813, K50.812, K50.818, K50.80, K50.811, K50.819, K50.1, K50.114, K50.113, K50.112, K50.118, K50.10, K50.111, K50.119, K50.0, K50.014, K50.013, K50.012, K50.018, K50.00, K50.011, K50.019, K50, K50.914, K50.913, K50.912, K50.918, K50.90, K50.911, K50.919
Chronic Infectious Disease	V08, Z21, 042, B20, O98.72, O98.711, O98.712, O98.713, 043, 043.3, 079.53, B97.35, B16, B16.1, B16.9, B16.2, B17.1, 070.41, B17.11, B17.10, 070.51, Z22.51, Z22.52, 070.44, 070.54, B18.0, B18.1, B18.2, V02.61, V02.62, B19.10, 070.71, B19.21, 070.70, B19.20, 070.2, 070.21, 070.20, 070.23, 070.22, 070.31, 070.30, 070.33, 070.32, 070.52, 070.42
Multiples Gestation	662.3, 662.31, O63.2, 662.30, 660.53, 660.51, 660.50, V91.03, V91.02, V91.01, V91.09, V91.00, 651.0, 651.03, 651.01, O30.041, O30.042, O30.043, O30.049, O30.031, O30.032, O30.033, O30.039, O30.011, O30.012, O30.013, O30.019, O30.0, O30.091, O30.093, 651.00, O30.001, O30.002, O30.003, O30.009, 651.33, 651.31, 651.30, 651.70, O30.91, O30.92, O30.93, O30.90, 652.63, 652.61, O31.8X21, O31.8X22, O31.8X31, O31.8X32, O31.8X30, O31.8X91, O31.8X99, 651.83, 651.81, 651.80, O30.809, V91.90, V91.91, 651.93, 651.91, 651.90, 651.73, 651.71, V91.20, V91.22, V91.21, V91.19, V91.10, V91.12, V91.11, 651.1, 651.13, 651.11, O30.1, O30.191, O30.192, 651.10, O30.101, O30.102, O30.103, O30.109, 651.43, 651.41, 651.40, O30.121, O30.122, O30.123, O30.111, O30.112, O30.113, O30.119, 651.2, 651.23, 651.21, O30.2, 651.20, 651.53, 651.51
STI During Pregnancy	094.3, A52.2, 093.9, A52.00, 090, A50, 090.9, A50.9, 090.1, A50.1, 090.0, A50.0, 090.2, A50.2, A51,

	091.81, 091.4, 091.0, 092, A51.5, 092.0, 092.9, 091.89, 091.2, 091.1, 091.3, 091.62, 091.61, 091.82, 091.51, 091.52, A51.9, 795.6, 090.40, 090.6, A50.6, 090.7, A50.7, 097.1, A53.0, 095.9, A52, 096, A52.8, 097.0, A52.9, 647.03, 647.00, 647.01, 647.04, 094, 094.9, A52.3, 104.0, 090.49, 090.5, A50.5, A50.59, A51.39, 093.89, 095.8, 094.89, A52.19, A51.1, A51.0, A51.2, 091.7, O98.12, O98.111, O98.112, O98.113, O98.119, 095.5, A52.77, 095.4, 095.3, A52.74, 095.1, 095.6, 095.7, 097.9, A53.9, 091.9, V08, Z21, 042, B20, O98.72, O98.711, O98.712, O98.713, O98.719, 079.53, B97.35, A56.01, A56.11, A56.3, A56.2, A56.0, A56.00, A74.9, A56.02, 099.52, 099.53, 099.54, 099.59, 099.56, 099.51, 099.55, 099.50, 099.41, A56.19, A56.09, 079.88, 078.88, 079.8, A56.8, 079.98, 079.9, 098.30, 098.50, A54.42, 098.52, 098.15, 098.35, A54.03, 098.40, 098.11, A54.01, 098.31, 098.16, 098.36, 098.42, A54.24, A54, 098.0, 098.10, 098.7, A54.6, A54.00, A54.49, 098.89, 098.6, 098.2, A54.9, 098.81, 098.82, A54.81, 098.86, A54.85, A54.22, 098.12, 098.32, 098.37, 098.17, A54.86, 098.53, 098.51, A54.02, O98.22, O98.211, O98.212, O98.213, O98.219, O98.23, 647.13, 647.10, 647.14, 647.11, 647.12, 098.49, 098.59, A54.09, A54.8, A54.89, 098.19, B16.1, B16.9, B16.2, B17.1, 070.41, B17.11, B17.10, 070.51, Z22.51, Z22.52, 070.44, 070.54, B18.0, B18.1, B18.2, V02.61, V02.62, 573.2, 573.1, B19.10, 070.71, B19.21, 070.70, B19.20, 070.2, 070.21, 070.20, 070.23, 070.22, 070.31, 070.30, 070.33, 070.32, O98.42, O98.411, O98.412, O98.413, O98.419, O98.43
Gestational Diabetes	O24.420, O24.424, O24.42, O24.429, O24.410, O24.414, O24.41, O24.419, O24.430, O24.439, O24.4, 648.03
Gestational HTN/Preeclampsia	O13.1, O13, O13.2, O13.3, O13.9, 642, 642.7, 642.73, 642.70, 642.74, 642.71, 642.72, 642.34, 642.30, 642.31, 642.32, 642.93, 642.9, 642.90, 642.94, 642.91, 642.92, 642.63, 642.60, O15.1, O15.02, O15.03, O15.00, O15.2, O15, 642.64, O15.9, 642.61, 642.62, 642.43, 642.44, 642.40, 642.41, 642.42, O14.02, O14.03, O14.00, O14, O11.2, O11.3, O11.9, 642.5, 642.53, O14.1, 642.54, O14.12, O14.13, 642.50, O14.10, 642.51, 642.52, O14.92, O14.93, O14.90
Preterm Labor	O60, O60.12X1, O60.12X2, O60.12X3, O60.12X5, O60.12X0, O60.13X1, O60.13X2, O60.13X0, O60.14X1, O60.14X2, O60.14X0, O60.14, O60.0, O60.02, O60.03, O60.00, O60.10X1, O60.10X0, O42.112, O42.113, O42.119, O42.011, O42.012, O42.013, O42.019, O42.912, O42.913, O42.919, O60.23X0, 658.1, 658.13, 658.11, 658.10, O42, O42.10, O42.00, O42.90, 644.03, 644.00
Short Cervix	622.5, N88.3, 654.53, 654.51, 654.52, 654.54, 654.50, 618.81, N81.82, O34.31, O34.32, O34.33, O34.30
Chorioamnionitis	O41.1211, O41.1210, O41.12, O41.1221, O41.1222, O41.1223, O41.1220, O41.1231, O41.1232, O41.1233, O41.1230, O41.1239, O41.1291, O41.1290, 658.43, 658.41, 658.40, O41.1020
Endometritis	O86.12, 098.16, 098.36, 670.1, 670.12, 670.14, 670.10
Pyelonephritis	590.1, 590.11, 590.10, 590.8, 590.81, 590.80
Mastitis/Breast Abscess	675.23, O91.23, O91.212, O91.213, O91.219, O91.22, 675.22, 675.21, 675.24, 675.20, 675.13, 675.12, 675.11, 675.10, O91.13, O91.112, O91.113, O91.119, O91.12, 675.14, 675.83, 675.82, 675.80, 675.84, 675.92, 675.91, 675.94, 675.90
Intra-abdominal Abscess	567.38, K68.19, K68.1, 567, N73.2
Wound Infection	O90.0, 674.1, 674.14, 674.10, 674.12, 998.32, T81.31XA, T81.31XS, T81.31XD, 998.31, T81.32XA, T81.32XS, T81.32XD, 998.3, 998.30, T81.30XA, T81.30XS, T81.30XD, T81.30, 879.3, 879.2, 879.5, 879.4, 674.34, 674.30, 674.32, S31.103A, S31.103D, S31.603A
All-cause Sepsis	A41.50, A41.59, A41.8, A41.89, A40.8, 670.2, 670.22, O85, 670.24, 995.91, A41.4, A41.81, A41.51, A41.3, A41.02, A41.01, A41.1, A41.52, A41.53, A41.0, A40.0, A40.1, A40.3, A41.2, A41.9, 995.92, R65.2, R65.20, R65.21, A40, A40.9
